# Discrimination, depressive symptoms, and prescription opioid misuse among adults with chronic pain who engage in hazardous drinking

**DOI:** 10.1080/00952990.2025.2576709

**Published:** 2026-02-05

**Authors:** Victoria E. Carlin, Emma C. Lape, Alexa G. Deyo, Grant H. Ripley, Sarah E. Polhill, Emily L. Zale, Joon Kyung Nam, Michael J. Zvolensky, Stephen A. Maisto, Joseph W. Ditre

**Affiliations:** a Center for Health Behavior Research & Innovation, College of Arts & Sciences, Syracuse University, Syracuse, NY, USA; b Department of Psychology, College of Arts & Sciences, Syracuse University, Syracuse, NY, USA; c Department of Psychology, Harpur College of Arts & Sciences, Binghamton University, Binghamton, NY, USA; d Department of Psychology, University of Houston, Houston, TX, USA; e HEALTH Institute, University of Houston, Houston, TX, USA; f Department of Behavioral Science, The University of Texas MD Anderson Cancer Center, Houston, TX, USA

**Keywords:** Chronic pain, discrimination, everyday discrimination, opioids, depression, alcohol, comorbidity

## Abstract

**Background:**

Prescription opioid misuse (i.e. use without a prescription or in ways other than prescribed) is a significant health concern among individuals with chronic pain. Hazardous alcohol use (i.e. drinking that increases risk of negative consequences) is common among individuals with pain, including among those who are prescribed opioids. Everyday discrimination, which is characterized by interpersonal experiences of identity-based harassment, has been independently linked to both depressive symptoms and prescription opioid misuse. Although promising as a potentially modifiable intervention target, the mediating role of depressive symptoms in associations between everyday discrimination and prescription opioid misuse remain largely unexplored. Further, it is important to identify factors associated with prescription opioid misuse among individuals with chronic pain who engage in hazardous alcohol use, as both are positively associated with prescription opioid misuse.

**Objectives:**

To examine indirect associations between everyday discrimination and prescription opioid misuse via depressive symptoms among adults with chronic pain who engage in hazardous drinking.

**Methods:**

Participants included 150 adults with pain (35.7% Black/African American; 59.7% female; *M*_age_ = 44.27) who were prescribed opioids and drank hazardously.

**Results:**

A process model revealed that depressive symptoms acted as mediator of associations-between everyday discrimination and prescription opioid misuse (*b* = 0.26, bootstrapped 95% CI [0.15, 0.39]). Specifically, everyday discrimination was associated with greater depressive symptoms, which in turn was associated with greater prescription opioid misuse.

**Conclusion:**

These findings suggest that providers should screen for depressive symptoms in the context of prescription opioid misuse, particularly among individuals who experience discrimination.

Prescription opioid misuse, defined as taking opioid medication without a prescription or in a manner other than what is prescribed (1), represents a substantial public health challenge, as approximately 294,000 Americans died from overdoses involving prescription opioids between 1999–2022 (2). Individuals who experience chronic pain may face elevated risk for prescription opioid misuse. Indeed, opioid medications are often prescribed as chronic pain treatment, and a systematic review estimated that 22% of adults with chronic pain engaged in prescription opioid misuse, compared to 3% of the general population (3, 4). A growing body of literature has documented links between chronic pain and hazardous drinking (i.e., patterns of alcohol consumption that increase risk of adverse physical and/or psychosocial health outcomes (5, 6)), and evidence suggests that hazardous drinking and prescription opioid misuse often co-occur, particularly among individuals with chronic pain (7, 8), reflecting the importance of identifying correlates of prescription opioid misuse, particularly among individuals with pain who engage in hazardous alcohol use.

Discrimination is the unfair or inequitable treatment of an individual due to a devalued or stigmatized identity, such as race, ethnicity, gender, sexual orientation, religion, or disability status that manifests through both structural forces and interpersonal dynamics (9-11). Everyday discrimination refers to frequent, interpersonal experiences of harassment or unfair treatment due to a stigmatized identity (12). Over time, exposure to instances of mistreatment can elevate allostatic load, or the physiological dysregulation that accumulates due to chronic stress (13, 14), ultimately contributing to poor physical and mental health outcomes (15-17). Indeed, the stress process model posits that exposure to stress leads to deleterious mental health outcomes (18), and highlights the importance of social context in shaping exposure to stressful experiences (19). Individuals who experience everyday discrimination may use substances to cope with accompanying stress and negative affect (20), and an accumulating body of research links everyday discrimination to various forms of substance use (21-23), including prescription opioid misuse (24-26). Notably, everyday discrimination has been implicated in both the onset and severity of chronic pain (27-31), and hazardous alcohol use (21-23). Collectively, these findings highlight the importance of examining psychosocial consequences of everyday discrimination, particularly among individuals with chronic pain who engage in hazardous drinking

Depressive symptoms are consistently elevated among individuals who experience everyday discrimination (32-38), and prior research indicates that depressive symptoms may follow exposure to discriminatory experiences (39). Depressive symptoms have also been implicated in prescription opioid misuse, as they have been shown to increase the likelihood of relapse following treatment for opioid use disorder (40) and are a predictor of initiating prescription opioid misuse (41, 42). Previous findings have demonstrated indirect associations between everyday discrimination, depressive symptoms, and the use of other substances (i.e., alcohol, tobacco, and cannabis (43)), providing initial empirical support for depressive symptoms as a potential psychological factor underlying associations between everyday discrimination and prescription opioid misuse. Given that they have been shown to play a role in prescription opioid misuse (40-42), chronic pain (44), and hazardous drinking ((45), depressive symptoms may represent a useful target of interventions addressing prescription opioid misuse among individuals with pain who engage in hazardous drinking patterns. Therefore, the goal of the current analysis was to test whether everyday discrimination is indirectly associated with prescription opioid misuse via depressive symptoms among a sample of adults with chronic pain who engage in hazardous drinking. Specifically, we hypothesized that everyday discrimination would be positively and indirectly associated with indices of opioid misuse via depressive symptoms.

## Materials and methods

### Participants and procedure

Participants included 150 adults who were recruited via online advertisements (i.e., Facebook and Craigslist ads) to participate in a web-based randomized controlled trial aimed at addressing co-use of alcohol and prescription opioids (NCT04592978). Participants completed baseline questionnaires and were then randomized to one of two conditions (1): an integrated pain-alcohol-opioid personalized feedback intervention, or (2) a control feedback intervention addressing pain and exercise/nutrition. Inclusion criteria for the parent study were as follows (1): adults aged 21 and above with chronic pain (2), current opioid prescription, and (3) above-threshold score on a measure of hazardous alcohol consumption (AUDIT-Consumption (45)). Although the parent RCT included assessments at base-line, posttest, and at 3-month follow-up, the present analyses include solely baseline data, prior to randomization or intervention. All procedures were approved by the Institutional Review Board (IRB) at Syracuse University, and all participants provided informed consent.

### Measurements

#### Prescription opioid misuse

The Current Opioid Misuse Measure (COMM (46)); assesses behaviors associated with opioid misuse using 17 items scored on a 4-point scale ranging from 0 (never) to 4 (very often). Scores were summed to compute a composite score reflecting the degree to which individuals engaged in behaviors related to opioid misuse over the past 30 days. The COMM has demonstrated strong diagnostic performance, including among individuals with chronic pain, and has been validated against similar measures of opioid misuse (47-50). The COMM exhibited excellent internal consistency in the current sample (α = 0.92).

#### Everyday discrimination

The short form of the Everyday Discrimination Scale (EDS (51)); is a widely used measure of the degree to which an individual experiences prejudiced or discriminatory treatment. It includes 5 items, each scored on a 6-point scale ranging from 1 (never) to 6 (almost every day), that assesses aspects of discriminatory experiences, such as “you are treated with less courtesy or respect than other people.” Items are summed to generate a total score ranging from 5–30, with higher scores reflecting more frequent discriminatory experiences. Notably, high EDS scores do not necessarily reflect discrimination experienced every day. After completing the main EDS items, individuals who endorsed any discriminatory treatment (e.g., responses of “less than once a year” or more frequently on any item) were asked “what do you think is the main reason for these experiences,” with response options such as “Your Ancestry or National Origins,” “Your Gender,” or “Your Race.” Although this item requests a single attribution for discriminatory treatment, the EDS measures discrimination more broadly, not necessarily tied to a single reason. The scale demonstrated good internal consistency among the current sample (α = 0.85).

#### Depressive symptoms

The 2-item Patient Health Questionnaire (PHQ (52)); measured depressive symptoms by assessing frequency of the following symptoms over the past 2 weeks: “little interest or pleasure in doing things” and “feeling, down, depressed, or hopeless.” Responses were scored on a scale ranging from 0 (never) to 3 (nearly every day), which were then summed to generate a total PHQ score ranging from 0–6, with higher scores reflecting more severe depressive symptoms. The PHQ has been validated as a measure of depressive symptom severity (52) and demonstrated good internal consistency in the current sample, as indicated by a Pearson correlation coefficient of *r* = 0.81.

#### Alcohol use

The Alcohol Use Disorder Identification Test (AUDIT-Total (53)) consists of 10 items, summed to generate a Total score ranging from 0 to 40, with higher total scores reflecting more hazardous drinking behaviors. The AUDIT-Consumption (AUDIT-C) subscale assesses levels of alcohol consumption and has been widely used as a screening tool for hazardous drinking (45, 54, 55). The AUDIT-C was used to screen for hazardous drinking, with all participants scoring at or above established thresholds (3 for women and 4 for men (45)). The AUDIT-Total was administered as part of the study measures, and demonstrated good internal consistency in the current sample (α = 0.87).

#### Chronic pain

Characteristic pain intensity and pain-related disability were assessed using the Graded Chronic Pain Scale (GCPS (56)). Characteristic pain intensity was measured with three items (e.g., “In the last 3 months, ON AVERAGE, how would you rate your pain?”), and pain-related disability was measured with four items (e.g. “In the last 3 months, how much has pain interfered with your DAILY ACTIVITIES?”). These subscales were combined, using threshold scores on both subscales, to compute a categorical pain grade (I–IV), which reflects increasing levels of chronic pain severity and disability, with Grade I indicating low-intensity pain with minimal disability and Grade IV indicating high-intensity pain with severe disability. Internal consistency was acceptable for the GCPS Characteristic Pain Intensity score (*α* = 0.74) and excellent for the GCPS Pain-Related Disability score (*α* = 0.95).

### Data analytic plan

Analyses were conducted using IBM SPSS for Statistics Version 27 and PROCESS macro for SPSS (57). First, descriptive statistics and bivariate correlations were computed for all variables of interest. Next, assumptions were tested, and examination of standardized residuals and predicted values indicated violations in heteroskedasticity. Thus, a heteroskedasticity-consistent standard error and covariance matrix estimator (HC3) was utilized, in line with recommendations for ordinary least squares (OLS) regression (58, 59). No violations of linearity, normality, independence of errors, or multi-collinearity were observed. The primary analysis examined indirect associations between everyday discrimination and prescription opioid misuse via depressive symptoms using a process model (PROCESS Model 4 (57)). Everyday discrimination scores (EDS) were included as a predictor, depressive symptoms (PHQ) as the indirect effect variable, and current opioid misuse (COMM) as the outcome variable. GCPS pain grade and AUDIT-Total scores were included as covariates in the process model, to test the unique contribution of everyday discrimination and depressive symptoms on prescription opioid misuse, while accounting for previously observed associations with pain severity and hazardous drinking (7, 21-23, 42, 44, 60-64). Direct, indirect, and total effects were estimated, with the indirect effect considered statistically significant if the 95% confidence interval (CI) does not cross zero (57). Further, the proportion mediated statistic was calculated (i.e., the indirect effect divided by the total effect) to examine the proportion of variance in EDS and COMM scores were explained by PHQ score. Bootstrapping with 10,000 resamples was employed to generate path coefficients and a percentile-based bootstrap confidence interval (CI), addressing limitations in statistical power and minimizing Type 1 error rates (65).

## Results

### Participant characteristics

Participants included *N* = 150 individuals with chronic pain who were prescribed opioids and reported moderate to heavy drinking (61.3% female; *M*_age_ = 44.57; see Table 1). Approximately half (56.7%) of participants identified as White, and about a third (34.0%) identified as Black or African American. The sample was generally well educated, with 54.7% reporting having attained a bachelor’s degree or higher. Most participants (90.0%) endorsed any discriminatory experiences. The average EDS score was 13.59, suggesting experiences of discriminatory treatment around a few times a month. Participants reported various sources of discrimination on the EDS, with the most common being “Other” (16.0%) and “Your Race” (14.7%, see Table 2). The average COMM score was 16.12, which is indicative of behaviors consistent with prescription opioid misuse. Participants also endorsed moderate to high levels of chronic pain, with 79.3% endorsing a GCPS pain grade of 3 or 4, indicating high disability and moderately to severely limiting pain intensity. The average AUDIT-Total score among participants was 13.80, indicating drinking patterns associated with elevated risk for negative consequences.

### Bivariate correlations

Bivariate correlations between the primary variables (EDS score, COMM score, PHQ score) and covariates (GCPS pain grade, AUDIT-Total score) were examined (see Table 3). Higher EDS scores were correlated with higher COMM scores, PHQ scores, and AUDIT-Total scores.

### Indirect association between everyday discrimination and opioid misuse via depressive symptoms

**Figure 1.**
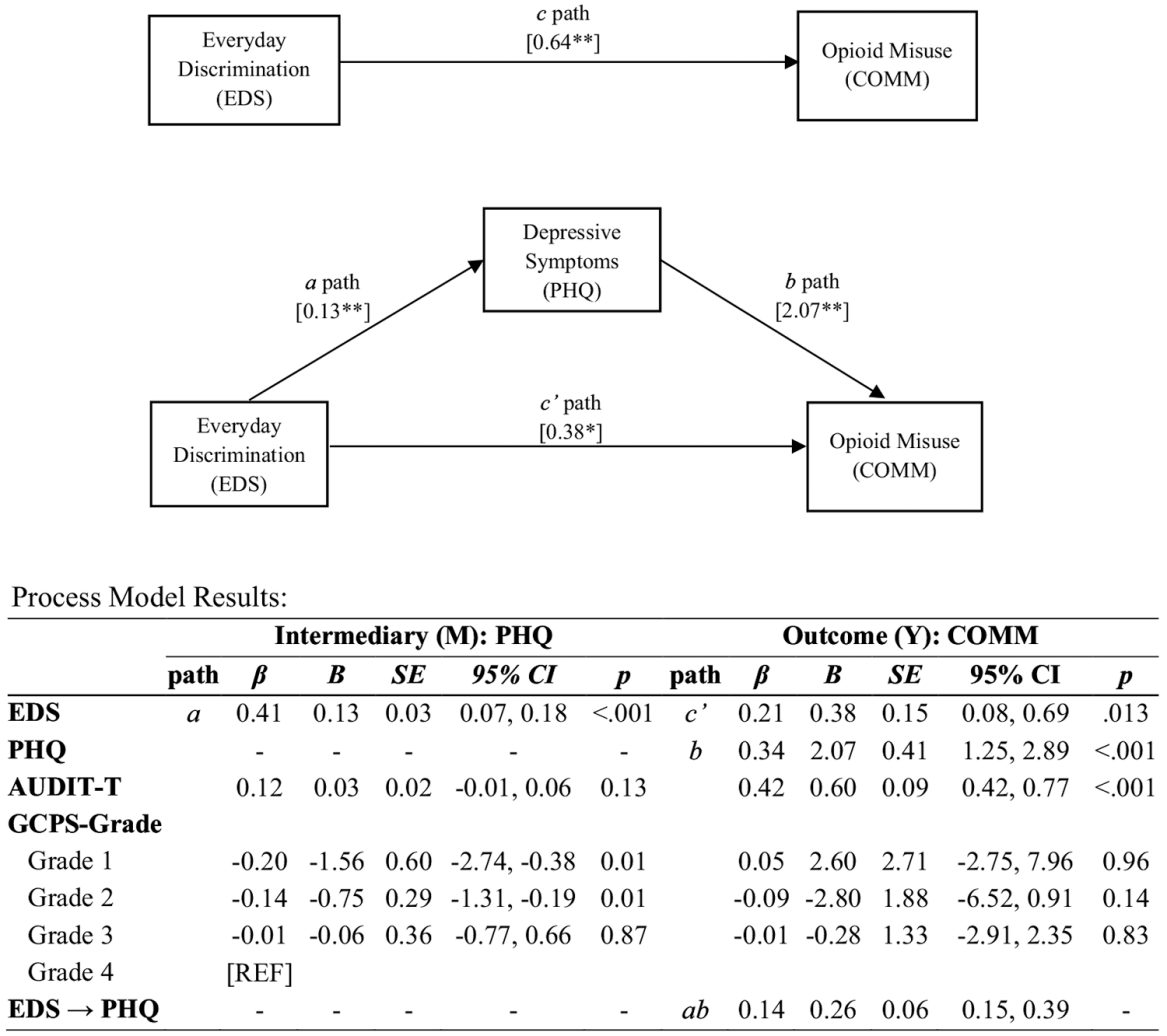
Indirect association between everyday discrimination (EDS) and prescription opioid misuse (COMM) via depressive symptoms (PHQ). *Note*: A heteroscedasticity consistent standard error and covariance matrix estimator (HC3) was used; *β* = standardized coefficient, *b* = unstandardized coefficient; EDS → PHQ is the indirect effect; The following covariates were adjusted for in this model: pain severity (GCPS Pain grade) and alcohol use (AUDIT-Total score); EDS – Everyday Discrimination Scale; PHQ – Patient Health Questionnaire; COMM – Current Opioid Misuse Measure; *p* < .01, *p* < .001.

Direct and indirect associations between EDS and COMM scores via PHQ scores were examined using a process model (PROCESS model 4 (57)), with the total effects model indicating a positive association between EDS and COMM scores (*b* = 0.64, bootstrapped 95% CI [0.33, 0.96], *p* < .01; *c* path). Additionally, there was a statistically significant indirect association between EDS and COMM scores via PHQ scores (*b* = 0.26, bootstrapped 95% CI [0.15, 0.39]). Specifically, EDS score was associated with greater PHQ scores, which, in turn, were associated with higher COMM scores. Further, the proportion of variation in the association between EDS and COMM scores explained by PHQ score was 40.2%, as indicated by the proportion mediated statistic. The direct association between EDS and COMM remained significant after accounting for the indirect association (*b* = 0.38, bootstrapped 95% CI [0.08, 0.69], *p* < .01; see Figure 1).

## Discussion

This is the first study to examine indirect associations between everyday discrimination and prescription opioid misuse via depressive symptoms among individuals with chronic pain who engage in hazardous drinking. Results indicated that everyday discrimination was positively and indirectly associated with prescription opioid misuse via depressive symptoms. This relationship was observed after accounting for pain severity and hazardous drinking, which are known correlates of everyday discrimination, depressive symptoms and prescription opioid misuse (7, 21-23, 42, 44, 61-64). Indeed, consistent with prior research (7, 66), results indicated that pain severity was positively associated with depressive symptoms, and hazardous drinking was associated with greater prescription opioid misuse.

The current results are broadly consistent with the stress process model, which posits that exposure to chronic stress leads to deleterious mental health outcomes (18, 19). Indeed, repeated experiences of discrimination produces chronic stress and depletes psychological coping resources (67), which may lead to depressive symptoms (37) and maladaptive coping behaviors such as opioid misuse (68). These findings also align with a conceptual model of allostatic load, or the physiological dysregulation that accumulates due to chronic exposure to stressors (13, 14). An established reciprocal model further posits that chronic pain and substance use interact as a positive feedback loop (69), such that pain motivates substance use, which in turn contributes to increases in allostatic load, ultimately increasing chronic pain severity (13, 70). It is possible that discrimination-related elevations in allostatic load may exacerbate this feedback loop, resulting in a greater risk for prescription opioid misuse among individuals with chronic pain.

These results also align with previous research showing positive covariation between everyday discrimination and prescription opioid misuse (24-26), and identify depressive symptoms as a potential pathway by which everyday discrimination may contribute to prescription opioid misuse. Notably, participants included individuals with chronic pain who engage in hazardous drinking – a group that may face an increased risk of both prescription opioid misuse and associated negative outcomes. For example, it has been estimated that alcohol contributes to 15% of deaths due to opioid overdose (71), and previous work has documented links between chronic pain and alcohol use (6, 69), including elevated rates of hazardous drinking among individuals with chronic pain (13, 72), experimentally induced pain as a motivator of drinking (73-75), and associations between pain severity and hazardous drinking (6, 69, 76). Similarly, while prior research in younger samples (e.g (77)., has demonstrated mediation of discrimination-substance use relations via depressive symptoms, our findings extend this work to a population in which substance use may be driven as much by pain and healthcare interactions as by social or affective factors. Unlike alcohol, tobacco, or cannabis, prescription opioid misuse among adults with chronic pain often occurs in the context of medical care and is shaped by factors such as pain management needs, prescription access, and stigma related to both pain and opioid use. Indeed, use of psychoactive substances may serve as a coping mechanism for discrimination-related stress and negative affect.

Although further research is needed to elucidate prospective links between everyday discrimination, depressive symptoms, and prescription opioid misuse, the current findings suggest that depressive symptoms may be an important target of prevention or intervention efforts (40-42). Interventions aimed at reducing opioid misuse may also be enhanced by concurrently addressing symptoms of depression, particularly among individuals from marginalized communities that tend to experience everyday discrimination more frequently. Furthermore, although these findings reflect the importance of integrated interventions that address chronic pain, mental health, and substance use concurrently, such integration is often complicated by structural barriers, limited provider training across disciplines, and resource constraints. Integrated interdisciplinary treatment models have shown greater success than parallel or siloed approaches (78), but more work is needed to reduce these barriers and to develop culturally tailored integrated interventions.

The current study has several limitations. First, these analyses are cross-sectional, and therefore do not necessarily reflect causal effects. Future work should examine prospective temporal associations between everyday discriminatory experiences and their influence on depressive symptoms and prescription opioid misuse. Methods such as ecological momentary assessment or longitudinal study designs could be leveraged to examine proximal antecedents of prescription opioid misuse or alcohol-opioid co-use among marginalized individuals with chronic pain. Second, this study employed a version of the everyday discrimination scale that did not allow participants to indicate multiple reasons for their experiences, which limited our ability to characterize perceived reason for discriminatory treatment among the current sample. This approach differs from existing literature, which often focuses on specific minoritized populations or forms of discriminatory treatment. Nonetheless, the current results broadly align with prior research demonstrating associations between everyday discrimination and adverse mental health and substance use outcomes among both racial and ethnic minoritized groups (24, 26, 32, 35, 37, 38), and sexual and gender minoritized groups (21, 22, 25, 39, 79). In addition, intersectionality research suggests that individuals who hold multiple marginalized identities are likely to experience overlapping forms of discrimination (79, 80), and qualitative research has shown that a substantial proportion of individuals report difficulty attributing their discrimination experiences to a single cause (81). Future research should seek to disentangle perceived reasons for discrimination, perhaps using an intersectional framework (80), to better understand and more effectively intervene on depressive symptoms stemming from discriminatory experiences. Third, the everyday discrimination scale does not capture structural sources of discrimination (e.g., wealth inequality, barriers to healthcare access), which likely contribute to the relations between everyday discrimination, depressive symptoms, and prescription opioid misuse. For example, people from racial and ethnic minoritized groups, who experience everyday discrimination more frequently (82), also face disparities in healthcare access and treatment (83-85), including less access to both opioid and non-opioid pain treatments (86-91). More research is needed to examine interplay between structural and interpersonal sources of discrimination, depressive symptoms and prescription opioid misuse. Fourth, participants in this study had chronic pain, were prescribed opioids, and engaged in hazardous drinking. Hazardous drinking may contribute to prescription opioid misuse, particularly in the context of chronic pain (7, 61-64, 92). It is possible that relations between everyday discrimination, depressive symptoms, and prescription opioid misuse could differ among individuals with no chronic pain or those who drink more intermittently. Fifth, depressive symptoms and prescription opioid misuse are hypothesized to have a reciprocal relationship, so it is possible that prescription opioid misuse may contribute to depressive symptoms (42). Likewise, it is possible that depressive symptoms may contribute to heightened perceptions of or reactions to everyday discrimination. Sixth, the direct association between everyday discrimination and prescription opioid misuse remained significant after accounting for the indirect effect of depressive symptoms. This finding suggests that, although depressive symptoms represent an important pathway, additional mechanisms such as social anxiety, somatic symptoms (39), or depletions in psychological coping resources (17) may also contribute to this relationship. Finally, the present analysis utilized a brief and unidimensional measure of depressive symptoms. Previous work has highlighted the multidimensionality of depressive symptomology, including psychological (i.e., anhedonia), cognitive (i.e., difficulty concentrating) and neurovegetative symptoms (i.e., sleep disturbances (93, 94). Future research should explore the unique contributions of the dimensions of different depressive symptoms in the indirect association between everyday discrimination and prescription opioid misuse.

In summary, the present results indicate that, among hazardous drinkers with chronic pain who are prescribed opioids, everyday discrimination is indirectly associated with greater prescription opioid misuse via depressive symptoms. These findings and future work may highlight the potential clinical utility of interventions that address depressive symptoms in the context of prescription opioid misuse, particularly among individuals who experience elevated levels of everyday discrimination.

## Figures and Tables

**Table 1.** Sociodemographic, discrimination, substance use, and pain characteristics.

	Total (*N* = 150)
	*n* (%)
Gender	
Female	92 (61.3%)
Race	
White	85 (56.7%)
Black or African American	51 (34.1%)
American Indian/Alaska Native	1 (0.7%)
Asian	2 (1.3%)
Multiracial/other	11 (7.3%)
Ethnicity	
Hispanic	22 (14.7%)
Income	
< $10,000	3 (2.0%)
$10,000–$25,000	21 (14.0%)
$25,000–$50,000	34 (22.7%)
$50,000–$75,000	43 (28.7%)
$75,000–$100,000	26 (17.3%)
> $100,000	23 (15.3%)
Education	
High school graduate/GED or less	14 (9.4%)
Some college	32 (21.3%)
Technical school/Associate’s degree	22 (14.7%)
4-year college degree	51 (34.0%)
School beyond 4-year college degree	31 (20.7%)
GCPS Grad^a^	
1 (Low intensity/low disability)	9 (6.0%)
2 (High intensity/low disability)	22 (14.7%)
3 (High disability/moderately limiting)	36 (24.0%)
4 (High disability/severely limiting)	83 (55.3%)
	*M* (*SD*)
Age	44.57 (13.12)
EDS Score^b^	13.59 (6.22)
PHQ-2 Score^c^	2.28 (1.89)
COMM Score^d^	16.12 (11.69)
AUDIT Score^e^	13.80 (8.34)

***Note***. ^a^Graded Chronic Pain Scale; ^b^Everyday Discrimination Scale; ^c^Patient Health Questionnaire—2 item measure of depressive symptoms; ^d^Current Opioid Misuse Measure; ^e^Alcohol Use Disorder Identification Test — Total score.

**Table 2.** Perceived main reason for discrimination.

	Total (*N* = 150)
	*n* (%)
Your Ancestry or National Origins	4 (2.7%)
Your Gender	11 (7.3%)
Your Race	22 (14.7%)
Your Age	11 (7.3%)
Your Religion	1 (0.7%)
Your Height	2 (1.3%)
Your Weight	10 (6.7%)
Some other Aspect of Your Physical Appearance	19 (12.7%)
Your Sexual Orientation	3 (2.0%)
Your Education or Income Level	3 (2.0%)
A physical disability	5 (3.3%)
Your shade of skin color (NSAL)	4 (2.7%)
Other	24 (16.0%)
Missing Response	
Reported no everyday discrimination	15 (10.0%)
Skipped question	16 (10.6%)

***Note.*** Frequency of responses to item 6 of the Everyday Discrimination Scale (i.e., “What do you think is the main reason for these experiences?”); Participants could only select one category.

**Table 3.** Bivariate correlations between variables of interest.

Variable	1	2	3	4	5
1. EDS^a^	—				
2. COMM^b^	0.56**	—			
3. PHQ^c^	0.48**	0.58**	—		
4. GCPS Pain Grade^d^	0.14	0.21*	0.28**	—	
5. Audit-Total^e^	0.41**	0.62**	0.32**	0.16	—

***Note***. ^a^Everyday Discrimination Scale; ^b^Current Opioid Misuse Measure; ^c^Patient Health Questionnaire— 2 item measure of depressive symptoms; ^d^Graded Chronic Pain Scale; ^e^Alcohol Use Disorders Identification Test; *p < .05. **p < .01.
